# 9th Hatter Biannual Meeting: position document on ischaemia/reperfusion injury, conditioning and the ten commandments of cardioprotection

**DOI:** 10.1007/s00395-016-0558-1

**Published:** 2016-05-10

**Authors:** R. M. Bell, H. E. Bøtker, R. D. Carr, S. M. Davidson, J. M. Downey, D. P. Dutka, G. Heusch, B. Ibanez, R. Macallister, C. Stoppe, M. Ovize, A. Redington, J. M. Walker, D. M. Yellon

**Affiliations:** The Hatter Cardiovascular Institute, Institute of Cardiovascular Science, University College London, 67 Chenies Mews, London, WC1E 6HX UK; Department of Cardiology, Aarhus University Hospital, Aarhus, Denmark; MSD A/S, Copenhagen V, Denmark; Department of Physiology, University of South Alabama College of Medicine, Mobile, AL USA; Department of Cardiovascular Medicine, Addenbrooke’s Hospital, Cambridge, UK; Institute for Pathophysiology, West German Heart and Vascular Center, University of Essen Medical School, Essen, Germany; Centro Nacional de Investigaciones Cardiovasculares Carlos III (CNIC), Madrid, Spain; Centre for Clinical Pharmacology, University College London, London, UK; Department of Anesthesiology, University Hospital Aachen, Aachen, Germany; Centre de recherche en Cancérologie de Lyon, Université Lyon, Lyon, France; Department of Pediatric Cardiology, the Heart Institute at Cincinnati Children’s Hospital, Cincinnati, OH USA

**Keywords:** Ischaemia, Reperfusion, Injury, Infarction, Pre-clinical, Basic research, Clinical trials, Ischaemic, Preconditioning, Postconditioning, Conditioning, RISK pathway, SAFE pathway, p2y12, Opiates, Asprin, Beta blockers, Statins, Metoprolol, Cyclosporine, CABG, Valve replacement, Cardiac surgery, Mitochondrial transition pore, Necrosis, Apoptosis, Necroptosis, Autophagy, Pyroptosis, DNA

## Abstract

In the 30 years since the original description of ischaemic preconditioning, understanding of the pathophysiology of ischaemia/reperfusion injury and concepts of cardioprotection have been revolutionised. In the same period of time, management of patients with coronary artery disease has also been transformed: coronary artery and valve surgery are now deemed routine with generally excellent outcomes, and the management of acute coronary syndromes has seen decade on decade reductions in cardiovascular mortality. Nonetheless, despite these improvements, cardiovascular disease and ischaemic heart disease in particular, remain the leading cause of death and a significant cause of long-term morbidity (with a concomitant increase in the incidence of heart failure) worldwide. The need for effective cardioprotective strategies has never been so pressing. However, despite unequivocal evidence of the existence of ischaemia/reperfusion in animal models providing a robust rationale for study in man, recent phase 3 clinical trials studying a variety of cardioprotective strategies in cardiac surgery and acute ST-elevation myocardial infarction have provided mixed results. The investigators meeting at the Hatter Cardiovascular Institute workshop describe the challenge of translating strong pre-clinical data into effective clinical intervention strategies in patients in whom effective medical therapy is already altering the pathophysiology of ischaemia/reperfusion injury—and lay out a clearly defined framework for future basic and clinical research to improve the chances of successful translation of strong pre-clinical interventions in man.

## Background

**Fig. 1:**
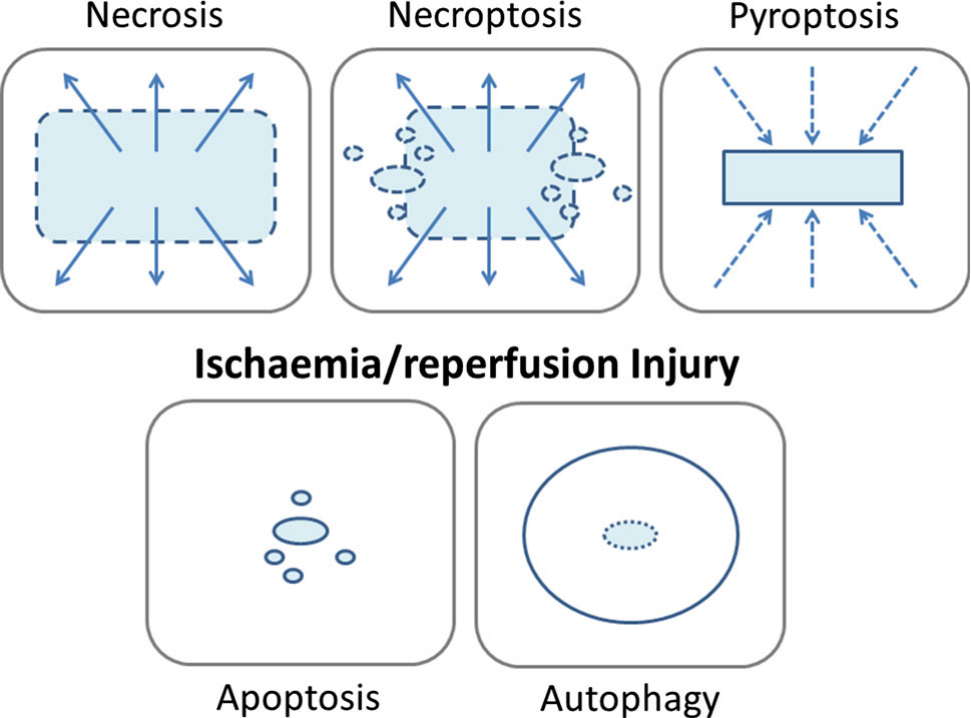
Cartoon of injurious ischaemia/reperfusion injury and the different forms of cell death. Necrosis is the prototypical form of cell death resulting from prolonged ischaemia. Through high-energy phosphate depletion, the cells cease to maintain electro-chemical gradients and the cells and the intracellular organelles swell. Histologically, the cytoplasmic membranes become progressively more lucent, before rupturing leading to the dispersal of cellular contents into the extracellular space (although the nuclei may persist). The cellular contents, including both nucleic and mitochondrial DNA, form damage associated molecular patterns (DAMPs); signals that are also released into the extracellular space by necroptosis. Sharing features with necrosis and programmed cell death, apoptosis, necroptosis involves the recruitment of cellular pathways (typically through receptor-interacting protein *kinase* (*RIPK*)), that may be activated through the dissipation of DAMPS from neighbouring necrosed cells. Like necrosis, but unlike apoptosis, the cell membrane does not remain intact, and may lead to the release of further DAMPS. The ensuing inflammatory reaction can then lead to pryoptosis—inflammatory cytokine mediated injury. The consequence of the spreading wave of dying cells, like toppling dominos, is likely responsible for the formation of the characteristic confluent myocardial infarct. Apoptosis, in contrast, is the ordered process of cell death, through the successful completion of an ordered cellular shut-down and compartmentalisation of potentially injurious cellular contents that prevents unintended injury to neighbouring cells. Autophagy plays a role in the house keeping of healthy cells, removing senescent proteins and organelles, such as mitochondria (mitophagy). During ischemia/reperfusion injury, autophagy may be a double-edged sword: while autophagy may remove terminally injured and dangerous organelles and oxidised proteins, contributing to energy recovery in reperfusion, excess autophagy may be linked to apoptosis and excessive substrate degradation

**Fig. 2:**
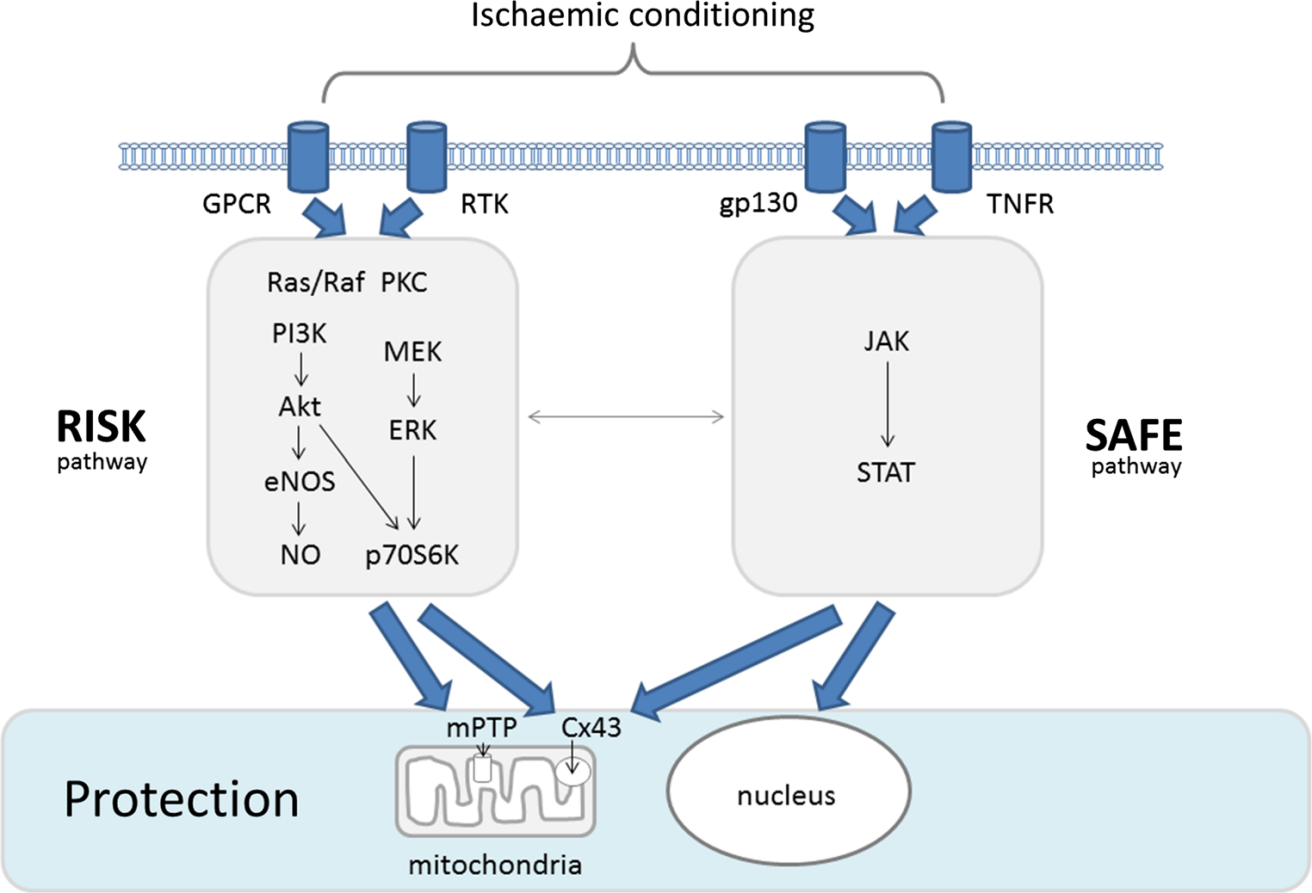
Reperfusion Injury Salvage Kinase (RISK) and Survivor Activating Factor Enhancement (SAFE) pathway model of ischaemic conditioning. The acute ischaemic stress of non-injurious ischaemia leads to the release of multiple stress-inducible factors that may activate through G-protein coupled receptors (GCPR) or receptor tyrosine kinases (RTK) to induce the RISK cascade, or through inflammatory cytokines via the glycoprotein 130 (gp130) or tumour necrosis factor receptor (TNFR) to activate the SAFE pathway. The resulting signalling cascade then impacts upon mitochondria, potentially inhibiting the mitochondrial permeability transition pore (mPTP) and other mitochondrial proteins such as connexin-43 (Cx43), or via the nucleus to induce, through promotors, new protein synthesis. *PI3K* (Phosphoinositide 3-kinase), *Akt* [Serine/threonine kinase (protein kinase B)], *eNOS* (Endothelial nitric oxide synthase), *ERK* (Extracellular signal-regulated kinases), *JAK* (Janus Kinase), *MEK* (Mitogen-activated protein kinase kinase), *NO* (Nitric oxide), *p70S6K* (p70 S6 ribosomal protein kinase), PKC (Protein Kinase C), *Ras/Raf* (small GTPase proteins), *STAT* (Signal transducer and activator of transcription)

Since the original description of ischaemic conditioning by Murry, Jennings and Reimer in 1986 [56], the understanding of the mechanisms of cell death arising from injurious ischaemia and reperfusion injury has been transformed: no longer a purely necrotic model, it is now recognised as a complex, multifaceted pathophysiological process [37], involving not only necrosis, but also cellular signalling, apoptosis, necroptosis [16] and the complex interaction of autophagy [15] through to inflammatory injury and pyroptosis [78] (Fig. 1). In parallel, identification of numerous pharmacological targets, both in modifying cell death pathways and in up-regulating canonical conditioning signalling Reperfusion Injury Salvage Kinase (RISK) [30] and Survivor Activating Factor Enhancement (SAFE) [48] pathways that culminate in the inhibition of the mitochondrial transition pore (mPTP, Fig. 2) have provided irrefutable proof of the existence of reperfusion injury following injurious ischaemia in animal models [32]. Moreover, the evolution of remote ischaemic conditioning the phenomenon whereby transient ischaemic stress of one organ can lead to protection of another, remote organ such as the heart against injurious ischaemia/reperfusion injury [33, 47] as a putative therapeutic intervention that can be applied prior to or immediately upon onset of reperfusion has supported the existence of ischaemia/reperfusion injury in man—both in proof-of-concept and meta-analysis of phase 2 clinical trials [46].

Over the concomitant period of time, clinical epidemiological data have clearly demonstrated what all practicing cardiologists already knew: the rates of cardiovascular mortality have been falling year-on-year over the last three decades [55, 64]—through a combination of social changes secondary to health education, improving primary and secondary prevention and improved management of acute coronary syndromes—not least through the introduction of primary percutaneous intervention (PCI) and optimised medical therapy. Nonetheless, while the efforts of cardioprotective strategies such as primary PCI have led to reduced early cardiovascular mortality, the “cardioprotection paradox” has been the incremental increase in the number of patients living with the consequence of myocardial injury: ischaemic cardiomyopathy and heart failure [10, 55]. Ischaemic injury is the leading aetiology of heart failure worldwide [55] and given that the propensity to develop heart failure is related to the extent of the primary myocardial injury [52], it is clear that further intervention to reduce the initial myocardial injury is not only desirable, but also necessary.

Ischaemic and pharmacological conditioning strategies are promising interventions for further improving outcomes, particularly for patients suffering from acute myocardial ischaemia/reperfusion injury resulting from ST-elevation myocardial infarction [69]. However, over the last 12 months, a number of phase 3 clinical trials studying cardioprotective modalities in a variety of clinical settings have been published, the results of which have not been universally positive in demonstrating the anticipated benefits in cardiovascular outcome.

## Remote ischaemic conditioning in cardiac surgery

Two recent large clinical outcome studies investigating the role of remote ischaemic preconditioning in cardiac surgery have been contemporaneously published in the New England Journal of Medicine. Remote Ischaemic Preconditioning for Heart Surgery (RIPHeart) [54] and Effect of Remote Ischaemic Preconditioning on Clinical Outcomes in CABG Surgery (ERICCA) [27]. Both of these studies sought to determine the efficacy of remote ischaemic conditioning (four cycles of 5 min upper limb ischaemia wrought by inflation of a blood pressure cuff to 200 mmHg and 5 min reperfusion with cuff deflation) in patients undergoing open-heart surgery and on-pump cardio-pulmonary bypass. With broadly similar primary end-points of death (any cause in RIPHeart, cardiovascular in ERICCA), rates of non-fatal MI and cerebrovascular accident, neither study was able to demonstrate a positive outcome for these measures. Curiously, in contrast to earlier clinical CABG trials, even differences in troponin release were not significantly different between control and active treatment groups. The reasons for the inability of these trials to reproduce the clear efficacy of basic and earlier, smaller clinical trials are unclear. One potential explanation may be an interval improvement in surgical and anaesthetic management protocols that has led to improved cardiovascular morbidity and mortality outcomes that has been observed over the last three decades [60, 66]. Indeed, recent innovations in surgical myocardial preservation techniques, such as combined antegrade and retrograde myocardial perfusion during bypass, are associated with smaller peri-operative myocardial injury [12]. Thus speculatively, optimisation of surgical and anaesthetic techniques may have led to a progressively smaller peri-procedural myocardial injury in patients undergoing CABG and valve surgery in recent years. With smaller peri-procedural injury, a type 1 statistical error is the likely consequence for studies in which power calculations are based on historical measures of myocardial injury and complications. Reduction of peri-procedural injury represents a genuine success for current surgical, anaesthetic and medical management strategies for the benefit of patients, but it also presents a diminishing target for additional benefit from conditioning-type cardioprotective interventions.

The converse argument is that current anaesthetic practice may be interfering with the cardioprotective mechanisms triggered by interventions such as remote ischaemic conditioning. Support for such a hypothesis can be found in comparable troponin release profiles in studies published over the last decade. Early, proof of concept trials consistently demonstrate an approximate 30 % reduction in the area under the troponin release profile curve over 48–72 h—with Troponin-T (TnT) peaks at 6–12 h consistently in the range of 700 ng/L in control patients. Interestingly, control patients in both RIPHeart and ERICCA have comparable TnT release profiles to these historical trials, but in contrast to the earlier studies, remote ischaemic preconditioning had lost its efficacy in reducing the release of this biomarker (ERICCA had a 10 % lower total troponin T release in patients who received remote ischemic preconditioning, an effect that was lost following multiple imputation analysis for missing data points). It has been argued that anaesthetic agents such as propofol may interfere with the canonical conditioning pathway [44] and more than 90 % of patients in ERICCA and all by protocol in RIPHeart, received propofol in preference to volatile anaesthesia. While the role of propofol is contentious (potentially cardioprotective in some settings, but largely neutral in CABG [62]) and biomarker release is not a clinical outcome, it may be relevant that absence of attenuation of troponin release by RIPC occurred in the two studies that did not demonstrate an outcome benefit, implicating a loss of biological effect [34]. Therefore, despite the neutral outcomes of RIPHeart and ERICCA, important questions remain unanswered in the context of cardiac surgery and the optimal anaesthetic management in the pre-, peri- and post-operative phases. Moreover, various peri-operative anaesthetic management strategies, from propofol anaesthesia to the administration of nitric oxide donors [e.g., intravenous glyceryl tri-nitrate (GTN) [42]; currently prospectively investigated in the ERIC-GTN trial [26]], require systematic careful investigation. In the presence of a cocktail of anaesthetic agents that may both inhibit canonical conditioning and are themselves cardioprotective, it is perhaps unsurprising that the demonstration of additional protection has become extremely difficult and perhaps also unnecessary. Moreover, there are concerns regarding both the nature of peri-procedural myocardial injury (i.e., how much is due to ischaemia/reperfusion injury versus direct mechanical tissue injury and perioperative inflammation) and the relevance of the relatively small release of troponin seen following cardiac surgery to clinical outcome has consequently cast doubt on the relevance of modern cardiac surgery as a model in which to test cardioprotective strategies, although the impact of conditioning strategies upon other post-surgical endpoints such as quality of life [22] has yet to be fully evaluated. The group considered that conditions that lead to greater myocardial injury (for example, acute ST-elevation myocardial infarction) would represent a better target for clinical study, with the potential for greater response to cardioprotective strategies in which to demonstrate efficacy.

Therefore, while further investigations into the myocardial benefit of remote ischaemic conditioning in cardiac surgery are not a high priority, it was felt by the group that close scrutiny and a more structured investigation into the optimal anaesthetic management of CABG patients is certainly warranted.

## Pharmacological cardioprotection in ST-elevation myocardial infarction

Two phase 3 clinical studies investigating the efficacy of two disparate pharmacological approaches in ST-elevation myocardial infarction (STEMI) patients were discussed: the Effect of METOprolol in cardioprotection During an Acute Myocardial Infarction (METOCARD-CNIC) [38] and Cyclosporine to ImpRove Clinical oUtcome in ST-elevation myocardial infarction patients (CIRCUS) [20] trials.

METOCARD-CNIC, a randomised, single-blinded, non-placebo-controlled clinical trial investigating the efficacy of metoprolol (a beta1-selective blocker) in attenuating infarct size in patients undergoing primary PCI for STEMI, was powered to demonstrate a 16 % reduction of infarct size with metoprolol administration compared to control, as measured by cardiac MRI. The study was positive, demonstrating a 20 % reduction of infarct size and concomitant biologically plausible improvements in secondary endpoints including ejection fraction and cardiac enzyme release. METOCARD-CNIC has been followed by the EARLY β-blocker administration before primary PCI in patients with ST-elevation myocardial infarction (EARLY-BAMI) study. Addressing the limitations of the earlier METOCARD-CNIC study, this is a randomised placebo-controlled, multi-centre, multinational trial but, at the time of the meeting, the study had not reported. There are further differences between the studies that may have an impact upon outcomes: EARLY-BAMI is recruiting patients with less restrictive inclusion criteria (infarcts of any location and up to 12 h after pain onset, compared to anterior STEMI only and 6 h in METOCARD-CNIC). The timing and dose of metoprolol administration in the 2 trials are also dissimilar: EARLY-BAMI has a 10 mg target dose of metoprolol (15 mg in METOCARD-CNIC), and the second bolus of medication is given immediately before reperfusion (in METOCARD-CNIC it was administered in the out of hospital setting long before reperfusion). However, the early METOCARD-CNIC data is promising, and while the mechanism of the protection following metoprolol administration is currently unknown, but may represent recruitment of a novel, non-canonical cardioprotective pathway that reflect known beneficial impacts upon inflammation [17] and arrhythmia [63, 75] and deserves further study. If proven it offers an interesting and useful inroad to modify ischaemia/reperfusion injury in addition to existing conditioning-mimetic strategies.

Inhibition of the mitochondrial permeability transition pore (mPTP) through pharmacological inhibition of cyclophilin-D is one such conditioning-mimetic strategy. The cyclophilin-D/mPTP is the putative end-effector of the canonical conditioning cardioprotective pathway which can be manipulated in the laboratory with cyclophilin inhibitors such as Cyclosporine and sanglifehrin-A. Cyclosporine-A appeared to be a promising candidate, with positive proof-of-concept clinical trial data [61] following a number of encouraging pre-clinical studies [29, 68] demonstrating the efficacy of such an approach. However, as has been widely reported elsewhere, the large clinical outcome study, CIRCUS, was neutral. Published commentaries have identified a number of potential explanations for the disappointing result and the apparent contradictory data when compared to earlier studies that include the formulation of the Cyclosporine (in CIRCUS, suspended in intralipid; intralipid itself having previously been shown to be independently cardioprotective [31, 50]). Other explanations were considered at the meeting to explain the differences between the pre-clinical and early proof-of-concept trials and CIRCUS, particularly in light of further neutral data from the recent multi-centre, randomised placebo-controlled, open-label CYCLE trial [59].

### Experimental versus clinical endpoints

Experimental studies concentrate on the need to reduce myocardial infarction, and predominantly concentrate on short-term reperfusion (hours to 1–3 days maximum) following the injurious ischaemic insult. In contrast, the key primary clinical outcome is patient survival following an acute myocardial infarction—be that at 30 or 365 days. It is not unreasonable to imagine that infarct size predicts mortality, and to an extent it does [52]—but other variables are at play, including ventricular remodelling and consequent heart failure [36] that are not modelled in short-term animal models. Extending pre-clinical animal studies to be inclusive of longer reperfusion times may provide additional, clinically relevant information [72].

### Type 1 error and overestimating benefit

Proof-of-concept trials perform a useful function, providing first-in-man evidence of a biological hypothesis, safety data and a platform on which to plan future investigations. What they are less capable of demonstrating is the genuine size of any therapeutic benefit in a “real-world” clinical setting—for which larger trials are necessary. Thus, positive proof-of-concept trials are interpreted with caution: they risk type 1 statistical (false positive) errors, tend to be undertaken in highly selected patient cohorts that make wider interpretation problematical, and where positive may over-estimate benefit of the study intervention. Consequently, the resuls may prove impossible to replicate in much larger, multi-centre/multi-national clinical trials with broader admission criteria which may dilute the effect of the cardioprotective intervention. Thus, while proof-of-concept trials continue to play an important role in providing scientific validity to a research question, outcome trials are necessary to demonstrate true clinical efficacy or lack thereof in the real-world—and both approaches continue to have their place.

### Is a single pharmacological targeted approach appropriate?

CIRCUS attempted to target one facet of the complex physiology of ischaemic conditioning: inhibition of cyclophilin-D and by extension, inhibition of mPTP opening. In fact, the mPTP is only partially inhibited even in the complete absence of cyclophillin-D (reviews [9, 25]). However, it is becoming increasingly apparent that cell death is not just mediated by a single channel in a single organelle. Signs of cellular injury are found throughout the injured cell: contractile apparatus, mitochondria, nucleus, sarcolemma, sarcoplasmic reticulum all demonstrate the characteristic hall-marks of fatal ischaemia/reperfusion injury. Thus targeting a single pathway may be naïve: the optimum intervention may, in fact, be the combination of multiple pathway targets in multiple intracellular compartments/organelles in multiple cell types that constitute the myocardium.

### Pharmacology and pharmacokinetics

STEMI represents a particular challenge for drug interventions designed to be cardioprotective. In order to imbue resistance against injurious ischaemia and reperfusion, the drug requires the necessary bioavailability at the appropriate site (myocardium) at the appropriate time. For drugs that are designed to be “anti-ischaemic”—impacting on the ischaemic period of the pathophysiological process of acute myocardial injury—any drug or remote conditioning signal needs to be able to permeate through into areas of the myocardium where blood flow will, at best, be extremely limited. Clinical trials investigating the potentially encouraging class of sodium/hydrogen exchange inhibitors (cariporide and eniporide in the GUARDIAN [70] and ESCAMI [77] studies, respectively) failed to demonstrate a significant reduction of myocardial necrosis in patients presenting with STEMI. This has been attributed to both the lack of access of these drugs to the ischaemic myocardium and the timing of their administration which was, out of necessity, relatively late into the injurious ischaemic insult, often just minutes prior to the onset of reperfusion. Indeed, pre-clinical data suggested that these drugs were only ever consistently cardioprotective when administered prior to the onset of injurious myocardial ischaemia, effectively negating their utility in the context of STEMI [1].

A similar problem of drug access also affects reperfusion-targeted therapy: while epicardial blood flow may prove to be radiologically excellent following PCI, there is no guarantee that microvascular flow will be preserved. Indeed evidence of microvascular obstruction (MVO) can be observed in approximately one-third of successfully revascularized patients. Ischaemia/reperfusion-mediated endothelial/microvascular injury and oedema may lead to limited access for cytoprotective drugs, impacting upon any potential beneficial effects one might hope to observe [35]. Moreover, the milieu into which these therapies are administered is made even more complex by interactions with concomitantly administered drugs. An example of this is the increasing recognition of the difficulties in obtaining adequate P2Y_12_ platelet receptor inhibition following the oral administration of direct, non-thienopyridine P2Y_12_ receptor inhibitors (e.g., ticagrelor) at the time of stent placement in primary PCI. Alterations of gut blood flow and attenuated absorption arise both as a direct consequence of the activation of the sympathetic/parasympathetic nervous systems following an acute myocardial infarct and as a result of the co-administration of opiate analgesics. Both significantly alter normal pharmacokinetics [45]. The optimal mode of administration of any cardioprotective therapy would, therefore, appear to be intravenous, but there are clear challenges in terms of understanding how such a drug reaches the ischaemic vulnerable zone in a timely and efficacious manner.

## Hurdles to successful translation

There are a number of valuable lessons that can be taken from the recent neutral cardioprotection trials that can be applied to future basic science study and subsequent clinical trial design to provide the best chance of successful translation of a potentially effective therapy.

### Animal models and reproducibility

In vitro, ex vivo and in vivo cell and animal studies remain the backbone for basic research to determine the mechanisms of cellular injury and to interrogate the cellular cytoprotective pathways and determine likely successful pharmacological targets that can be targeted to attenuate cell death in the face of injurious ischaemia/reperfusion injury. Data reproducibility is crucial. It is interesting to note that there was controversy regarding the likely efficacy of sodium-hydrogen exchange (NHE) inhibitors administered after the onset of ischaemia prior to the GUARDIAN and ESCAMI trials. Subsequently anxieties were also voiced regarding the likely efficacy of adenosine administration in this role prior to the largely neutral AMISTAD trials [18]. Even with Cyclosporine-A, it is unclear as to the drug’s efficacy in all species, with no infarct sparing in rats treated with Cyclosporine-A at the time of reperfusion in contrast to mice, and a meta-analysis finding no evidence for an effect on myocardial infarct size in swine [21, 51]. Therefore, it seems prudent to establish scientific consensus with appropriately powered, blinded and randomised pre-clinical animal trials prior to undertaking translation into man, as has been recommended previously [49, 65].

### Optimal clinical outcome identification

Basic research studies often concentrate on short-term reperfusion durations using infarct size as the primary end-point. There is a clear need for these studies, and they have a clear role in the development of hypotheses and delineation of mechanisms of both cell death and cellular salvage. However, in the pre-clinical translation pathway, it would be helpful to demonstrate efficacy of particular pharmaceutical interventions in animal models that perhaps more closely reflect the clinical end-points that physicians are striving to achieve in patients presenting with acute coronary syndromes—particularly mortality and the morbidity associated with loss of ventricular muscle, heart failure.

It would therefore seem prudent to demonstrate efficacy in reducing animal mortality and development of heart failure in longer-term experimental studies, extending at least until 30 days post-myocardial infarction.

### Optimum clinical target identification

Cardiac surgery has in many ways been an ideal clinical model of cardiac injury, with predictable onset of ischaemia and the restoration of flow following the completion of a successful bypass procedure. However, the extent of myocardial injury observed following cardiac surgery is significantly smaller than that seen following a presentation with STEMI. Consequently, with a smaller injury, the ability to detect a significant myocardial protective effect is diminished. Combined with surgical and anaesthetic improvements, it was felt by the group that it appears to be time for the cardioprotection field to concentrate upon areas of greatest clinical need with perhaps the greatest chance of demonstrating clinical benefit, i.e., acute myocardial infarction.

### The duration of ischaemia: too much of a bad thing

As already indicated, the duration of myocardial ischaemia has an important influence not only upon the extent of myocardial necrosis, but also upon the efficacy of a cardioprotective intervention. Very short durations of myocardial ischaemia may not result in a large enough injury to observe a measurable improvement in terms of myocardial salvage (and may even be exacerbated by interventions such as ischaemic postconditioning [53]). However very long durations of myocardial ischaemia not only result in greater ischaemia/reperfusion injury, but also have an impact upon the efficacy of the cardioprotective strategy being employed. [11, 24] Modelling clinically relevant ischaemia in small animals is difficult and extrapolation of durations of ischaemia from small animal models to humans is not direct. However, pre-clinical data demonstrate a clear failure of interventions such as ischaemic post-conditioning against more prolonged index ischaemia times [53] that needs to be considered in context of clinical trial design. Interestingly, the positive clinical outcomes in STEMI patients are in those patients with comparatively short ischaemic durations, being revascularised within 3–4 h from the onset of symptoms [43]. It is notable that patients recruited to the CIRCUS trial had a relatively long ischaemic time; 18 % had an ischaemic time of greater than 6 h.

### The dose of the conditioning stimulus: too much of a good thing

A comparatively under-studied potential pitfall of cardioprotective clinical studies is the dose of the pharmacological conditioning-mimetic or the number and duration of ischaemic conditioning cycles used in patients. Almost all cardioprotective drugs have dose–response curves with the classical “U”-shaped curve, which means more [drug] does not mean more [protection]. This drug–response characteristic is also evident with Cyclosporine-A, documented from its earliest characterisation [57]. Perhaps surprisingly, the same “U”-shaped dose–response relationship also exists with remote ischaemic conditioning: increasing the duration of remote limb ischaemia may ultimately lead to failure of the conditioning effect [41].

Unfortunately, the dose–response curves of many pharmacological agents and of remote ischaemic conditioning are not well characterised in man—and perhaps this is largely due to the lack of a clear biomarker of cardioprotection other than the attenuation of infarct size in patients presenting with acute myocardial injury. However, with remote ischaemic conditioning, the demonstration that it is possible to take a blood sample and dialyse the cardioprotective moiety and infuse this in a Langendorff-perfused rat heart [40, 67] may provide some useful insights into the minimum and maximum number of conditioning cycles required to imbue protection upon the heart and the potential impact of a variety of co-morbidities in patients who present with the sequelae of coronary artery disease.

### The influence of co-morbidities

Much has been written with respect to the adverse impact of co-morbidities upon the efficacy of ischaemic conditioning modalities [23]. In clinical practice, the critical co-morbidity is coronary artery disease: the final common pathophysiological pathway of the well-recognised cardiovascular risk factors of diabetes, hypertension and dyslipidaemia. In the sub-analysis of the CIRCUS study, diabetes, hypertension and dyslipidaemia had no incremental detrimental impact upon clinical outcome [20], and therefore it would seem that establishment of coronary artery disease itself is the critical process; the biological milieu appears not to be further worsened by the contributory disease states.

How the milieu of multiple co-morbidities impacts upon the efficacy of cardioprotective strategies is unclear. If the clinical outcome data can be used as a guide, it appears that multi-morbidity may be comparatively benign: the establishment of coronary artery disease may be the single co-morbidity that increases the conditioning threshold, a threshold that may not be further raised by the presence of other cardiovascular risk factors (e.g., diabetes, hypertension and hyperlipidaemia). This is an interesting hypothesis that requires further pre-clinical study.

### Improving clinical care: the success hypothesis

**Fig. 3:**
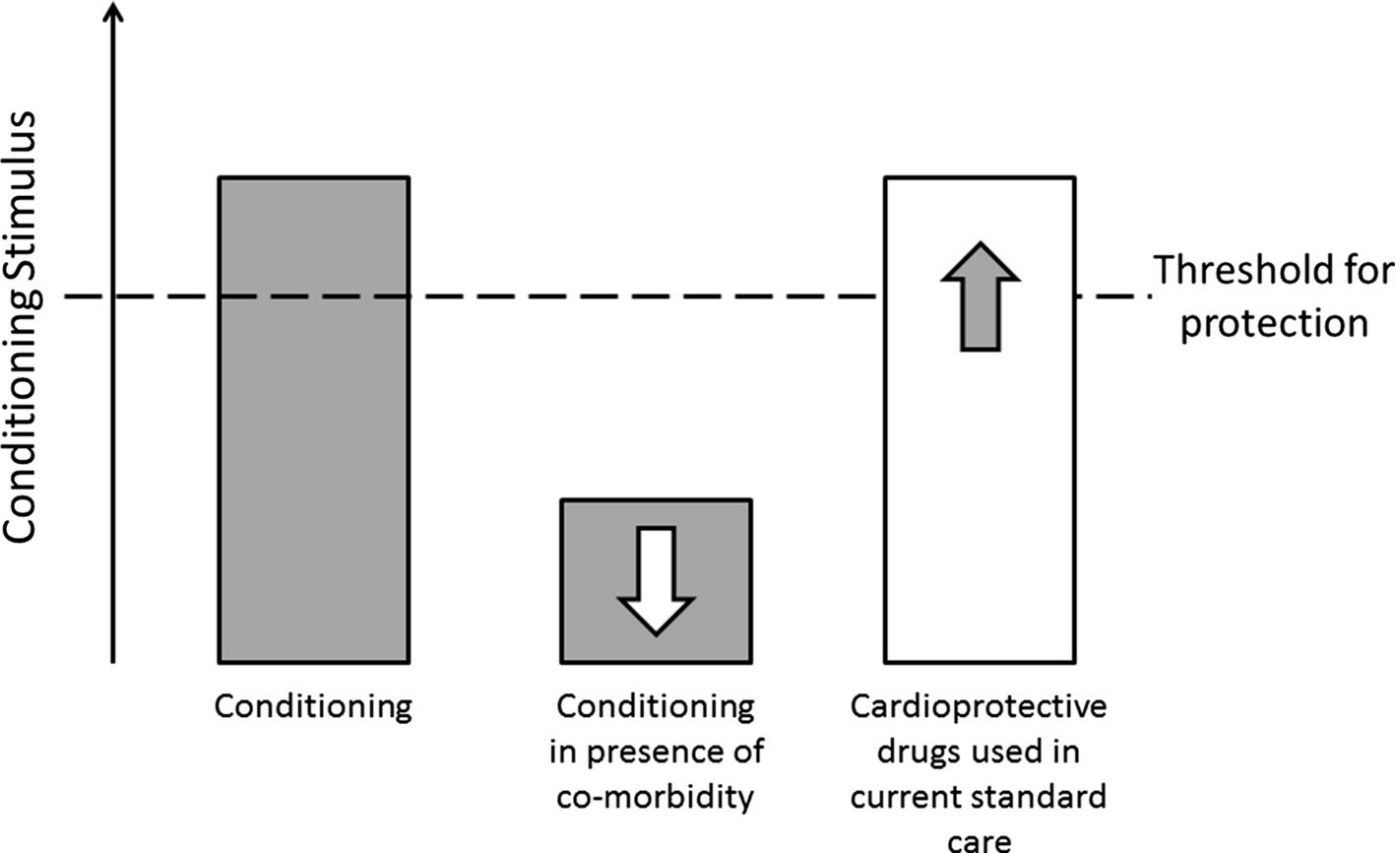
The conditioning stimulus and the impact of co-morbidity and drug therapies. In animal and human studies, it is possible to trigger cardioprotection with conditioning strategies—particularly if the subjects are young and free from co-morbidity. However, the efficacy of a conditioning stimulus is significantly blunted when there are co-morbidities presence (age, diabetes, hypertension, hypercholesterolaemia): the conditioning stimulus that was once effective, appears suppressed, and unable to exceed a critical threshold required for triggering protection. In contrast, with the “success hypothesis”, we find that medications already in regular use in patients presenting with acute coronary syndromes are already cardioprotective in their own right and likely already attenuating infarct size. Examples of these include opiates, P2Y_12_ inhibitors, statins, beta blockers, etc. (see *text*). Indeed, such a cocktail may be sufficient to trigger cardioprotection: the drugs have already exceeded the conditioning threshold, will reduce infarct size and optimise outcomes. While this makes the demonstration of efficacy of ischaemic conditioning-type strategies challenging, it represents a genuine benefit for patient outcomes. Further optimisation may, however, require targeting alternate mechanisms of cell injury

The improving mortality figures associated with cardiovascular disease is a genuine success story [64] for which all involved in the development and delivery of modern medical interventions should be commended. Modern practice has resulted in shorter ischaemic times wrought by the introduction of reliable revascularisation through primary PCI, but also reflects changes in medical management and pharmaceutical interventions. A typical patient presenting with a STEMI will receive a cocktail of medications on the way to, while in and following discharge from the coronary catheter laboratory. At the time of initial diagnosis, loading with aspirin and administration of opiate analgesics such as morphine will be administered by paramedic teams during transfer to the local heart attack centre. On arrival and confirmation of STEMI by medical staff, loading of a P2Y_12_ inhibitor such as ticagrelor, will proceed as the patient is transferred for the critical revascularisation procedure. During the procedure, heparin, bivalirudin or GpIIbIIIa inhibitors may be administered according to local practice—and on transfer to the coronary care unit, beta blockers, statins and angiotensin converting enzyme inhibitors will be initiated. While not all these therapies are cardioprotective, opiates [8], P2Y_12_ inhibitors [74], GpIIbIIIa inhibitors [3], beta blockers [39], statins [7] and ACE inhibitors [4] have each been demonstrated to reduce infarct size in basic science studies. Of these cardioprotective drugs, only opiates, P2Y_12_ and GpIIbIIIa inhibitors are reliably administered prior to the onset of reperfusion. While aspirin is also administered in an appropriate time window, there is no evidence to suggest that aspirin is cardioprotective [74]. Therefore, opiates, P2Y_12_ and GpIIbIIIa inhibitors, protective in the pre-clinical setting, have the potential to inadvertently recruit canonical conditioning pathways. Thus, through good medical practice, clinicians may already be successfully conditioning their patients and improving outcomes (see below and Fig. 3).

Future clinical trials need to be undertaken in the environment of these agents, but pre-clinical studies should also take cognisance of current clinical practice: as part of the bench to bedside translation pathway, animal studies ought be undertaken that reflect the current pharmacological milieu of acute coronary syndrome patients, and be shown to be effective on a background which includes drugs such as morphine and P2Y_12_ inhibitors.

### Novel cardioprotective strategies

P2Y_12_ inhibitors have already demonstrated significant improvements in cardiovascular mortality [71, 76]. P2Y_12_ inhibitors have a strong anti-infarct effect in animal models and the mechanism is dependent on the presence of platelets in the blood [6, 19]. There is evidence that they may protect by activating the canonical conditioning pathway: P2Y_12_-induced protection depends on similar signalling components as conditioning and adding ischemic postconditioning to the platelet inhibitor offered no additional protection to a rabbit heart [74]. Moreover, ticagrelor also inhibits adenosine re-uptake via the equilibrative nucleoside transporter, increasing adenosine and potentially triggering conditioning via this route [13, 58]. P_2_Y_12_ receptors are not restricted to platelets, however, and may also have impact upon inflammation (reviewed in [58]) that may suggest non-canonical mechanisms of protection. Given that myocardial injury is not restricted to the mitochondrion, other cardioprotective strategies that target a non-RISK/SAFE pathway may offer additional benefits to ischaemic or pharmacological conditioning. There are a number of novel interventions that have either clinical potential or have proven cardiovascular benefit. Matrix metalloproteinase (MMP) inhibition [5, 14] and prevention of mitochondrial DNA degradation [73] are examples of the former, and sodium/glucose transporter (SGLT) inhibition an example of the latter [79].

For MMP inhibition, pre-clinical data support a non-canonical cardioprotective pathway that appears independent of the RISK signalling pathway and cyclophilin-D mitochondrial permeability transition pore regulation [5]. Moreover, early open-label clinical trial demonstrate positive cardiovascular outcomes following administration of the MMP inhibitor, doxycycline [14]. MMP inhibition is not alone in demonstrating a non-canonical protective signature: inhibition of mitochondrial DNA degradation and release using novel delivery of endonuclease-III also represents a strong clinical target for the preservation of myocardial viability [73]. Interestingly, it was felt that it may be possible to apply a multi-modal approach to the diminution of myocardial injury: building additive cardioprotection from disparate cardioprotective interventions to maximise infarct limitation, which might be an attractive route to further improving cardiovascular outcomes in the current clinical paradigm.

SGLT inhibition has appeared virtually out of the blue as a remarkable new intervention for the management of type-2 diabetes. Developed as a therapeutic oral anti-hyperglycaemic agent through targeting of renal SGLT2 transport and promoting renal glucose clearance, the EMPA-REG study [79] demonstrated for the first time that a treatment for hyperglycaemia also has sizeable clinical benefits in terms of reducing not only blood pressure, but also cardiovascular mortality (without impacting on the frequency of myocardial infarction of treated patients). The mechanism by which this observation is mediated is unclear, but with pre-clinical data supporting a link between SGLT and reactive oxygen species generation [2], it is attractive to postulate that these drugs may have direct cardioprotective properties: a hypothesis that deserves further investigation.

## The ten commandments of cardioprotection

Recent clinical trial results have been disappointing, failing to deliver the anticipated patient benefit—but there is little doubt of the validity of ischaemia/reperfusion injury as a target for intervention in patients presenting with acute myocardial infarction. Further, large scale clinical trials—CONDI-2 and ERIC-PPCI [28], two concurrent and allied studies investigating remote ischaemic conditioning in patients presenting with STEMI—are currently recruiting, but recent data provides reason for reflection and consideration as to how best develop a clinical translation pathway so as to deliver clinical interventions with the best chance of delivering a positive clinical outcome for patients presenting with STEMI. The summary of the workshop contributors can thus be distilled down to the following ten points:

1. The cardioprotective intervention should be demonstrated in multiple models, that should ideally include large animals and models with co-morbidities, prior to clinical translation.
2. The data should be reproducible between laboratories (we propose the formation of an international laboratory consortium to ensure reproducibility of randomised blinded animal studies).
3. End-points in late pre-clinical studies should reflect the clinical endpoints that will influence clinical practice (to include mortality).
4. Pre-clinical translational studies should start to reflect the poly-pharmaceutical environments in which cardioprotective strategies will be employed in clinical trials—for example, on the background of current medical therapy for STEMI patients (e.g., opiates, P2Y_12_ inhibitors etc.).
5. Although hypothesis demonstrating (proof of concept) clinical studies have an important role in studying the mechanisms of cardioprotection in man, their outcomes require cautious interpretation in regard to changes in clinical practice.
6. Large scale, all-comer trials are required to definitely prove clinical benefit in patients presenting with STEMI. These trials should have clearly defined cardiovascular mortality and morbidity outcomes and be powered appropriately.
7. Reperfusion injury should henceforth be referred to as ischaemia/reperfusion injury to more accurately reflect the true nature of the pathophysiology, and the expected benefit of targeting both aspects.
8. Current management of CABG surgery is so good, that it may be difficult to demonstrate further cardioprotection. Therefore, CABG may not be a robust enough model for examining ischaemia/reperfusion injury: the success of current clinical practice has resulted in an inadequate target for cardioprotection.
9. Ischaemic conditioning is a powerful cardioprotective strategy in pre-clinical studies, but does not represent the sole cardioprotective pathway in the armamentarium against lethal ischaemia/reperfusion injury. Therefore, future cardioprotective strategies should aim to target multiple pathophysiological pathways for optimal protective benefit.
10. Remote ischaemic conditioning is a safe, cost-effective and non-invasive intervention that has been proven to be effective in a number of pre-clinical and clinical studies. A concerted effort is required to ascertain the mechanisms by which this protection occurs to maximise/optimise its potential benefits.
